# Phylogeny, Phytomedicines, Phytochemistry, Pharmacological Properties, and Toxicity of *Croton gratissimus* Burch (Euphorbiaceae)

**DOI:** 10.1155/2022/1238270

**Published:** 2022-05-17

**Authors:** Kirkland Dingili Magwilu, Joseph Mwanzia Nguta, Isaac Mapenay, Dorine Matara

**Affiliations:** Department of Public Health, Pharmacology and Toxicology, Faculty of Veterinary Medicine, University of Nairobi, Nairobi, Kenya

## Abstract

*Croton gratissimus* is an important plant in Africa setup and across the globe for its ethnomedicinal uses in managing a wide range of diseases. Its phylogeny, pharmacological properties, ethnomedicinal uses, phytochemistry, and cytotoxicity have been highlighted in various articles and journals. This review article aims to give a comprehensively overviewed literature about *Croton gratissimus* genus. Authentic literature sources such as books, peer reviewed articles, journals, theses, Google Scholar, Science Direct, and any other validated internet source have been used to develop this review. *Croton gratissimus* is richly found across different climatic zones because of its ability to adapt to various climatic conditions. It is mainly found in rocky hills as a scrub that is about 12–15 m tall. Its leaves are glossy, green on the top, and silvery underneath. Some of the leaves may look brick red rusty. *Croton gratissimus* has been explored traditionally to manage a number of diseases among the human race since time immemorial. It has been used to treat different ailments ranging from respiratory tract infections, urinary tract infections, malaria, diabetes, hypertension, dermatological conditions, arthritis, gastrointestinal disorders, fever, sexually transmitted diseases, and infertility. Studies have shown that parts of this plant have antioxidative, antimicrobial, anticholinesterases inhibitory, antidiabetic, antihyperlipidemic, anticonvulsant, antiulcer, antihypertensive, antiproliferative, antiplasmodial, and anti-inflammatory activities. Terpenoids and flavonoids have shown to be the major classes of compounds in this plant. Its toxicity has not been well established; some studies have suggested that *Croton gratissimus* can cause hepatotoxicity and genotoxicity. More studies are needed to elucidate the compounds and their structures giving this plant a wide range of pharmacological activities, efficacy, safety, and toxicity levels, since the plant has greater ethnomedicinal uses. This would give a great indication of discovering new novelties that can give a breakthrough in drug discovery.

## 1. Introduction

Traditional medicine has played a critical role in the health systems by providing alternative solutions to the well-being of both humans and animals. Various medicinal remedies have been used in the treatment and management of various ailments such as headaches, respiratory disorders, gastrointestinal disorders, sexually transmitted diseases, urinary tract infections, cardiac disorders, and snake bites among others [1]. These disorders have been successfully managed by plant products using different plant parts such as the stem, roots, bark of stems and roots, leaves, flowers, and fruits singly or a concoction of two or more parts of the plants [1]. Apart from being used as complimentary remedies for the management of ailments, plant products are used as nutraceuticals/phytonutrients, since they have rich reservoirs of minerals and vitamins contributing to various pharmacological activities [2–4].

In an era where antimicrobial resistance is of substantial concern across the globe, plant bioactives have provided promising alternatives medicines [5]. These bioactives have shown antiinfective activities across a range of microbes [6]. Despite scanty information about the efficacy and safety of complementary medicines of plant origin, herbalists and animals have continued to use plant products delivering effective treatment for various diseases [7, 8].

Antimicrobial drug resistance is a serious phenomenon of the 21^st^ century as a number of bacterial strains with multidrug resistance have emerged [9, 10]. It is a challenge for effective treatment of a wide range of infectious diseases caused by pathogens no longer susceptible to available treatment regimens [11]; hence, millions of people die every year as a result of antimicrobial resistance.

There are concerted efforts to develop new novelties to tackle antimicrobial resistance from plants and plants' products. Many researchers are conducting research on plant products to validate their antimicrobial effects [6]. Different plants and plant parts have shown to have phytochemicals with useful antimicrobial activity towards different microbial strains [12].

One of the plants that has shown tremendous potent bioactives is *Croton gratissimus*. *Croton gratissimus* belongs to the Euphorbiaceae family, which has many genera with *Croton* genus being one of them. *Croton* genus is believed to be the largest genus with about 1200–1300 species characterized by flowering plants. These plants include scrubs of various types, herbs, and trees. Due to richness in phytochemistry, numerous *Croton* species have been largely used in ethnomedicines in Africa [13]. It is widely believed to adapt to different climatic conditions, and this is probably the reason to be found across the globe in various climatic regions such as the tropics and subtropics.

Studies have shown *Croton gratissimus* has antioxidative effects against free scavenging radicals, neurogenerative agents, and cholinesterase inhibitory activities [14]. It has inhibited ovarian cancerous cell line growth through apoptosis [15, 16]. Anti-HIV [17] and anti-inflammatory effects have also been established [18, 19]. Its ethnomedicinal usefulness has been applied since time immemorial to treat respiratory disorders, malaria, diabetes, arthritis, hypertension, urinary tract infections, gastrointestinal disorders, and gonorrhea [20].

## 2. Phylogeny

### 2.1. Plant Description

This plant is referred to as lavender fever berry or lavender *Croton*. *Croton gratissimus* comes from the Greek words *Kroton* meaning tick (*Croton* seeds resemble a tick) and *gratissimus* means most pleasing (*gratus* = pleasing and *issimus* = most) [13]. It is a scrub of between 8 and 15 m tall and can grow to 20 m tall. It has glossy dark green leaves on the top and silvery to rusty coloured underneath. It has small, white to yellowish flowers in spikes about 10 cm long [21]. It has a grey and rough bark fissured near the base. The roots do not have the aggressive root system and therefore tolerate any soil type. It has a grouped, spiked flower, reddish to brown in colour. They are monoecious with males and females in separate flowers. The fruits are 3-lobed capsules about a centimeter wide, yellowish to brown at maturity. The seeds have a caruncle [22].

*Croton gratissimus* variety *gratissimus* and *Croton gratissimus* variety *subgratissimus* are the only two varieties of *Croton gratissimus*. The upper surface of variety *gratissimus* has no hairs, while variety *subgratissimus* contains stellate hairs [21].

*Croton gratissimus* has several synonyms such as “*Croton gratissimus* Burch, *Croton microbotryus* Pax, *Croton antunesii* Pax*, Croton welwitschianus* Müll. Arg, *Croton zambesicus* Müll. Arg, *Croton amabilis* Müll. Arg, *Oxydectes amabilis* (Müll. Arg.) Kuntze, *Oxydectes welwitschiana* (Müll. Arg.) Kuntze, and *Oxydectes zambesica* (Müll. Arg.) Kuntze” [22] (Table 1).

### 2.2. Taxonomy

  Kingdom: Plantae
  Phylum: Tracheophytes
  Class: Magnoliopsida
  Order: Malpighiales
  Family: Euphorbiaceae
  Genus: *Croton gratissimus*
  Variety: *Croton gratissimus* var. *gratissimus*; *Croton gratissimus* var. *subgratissimus*

### 2.3. Traditional Names

Different names of Croton gratissimus are given in Table 1.

## 3. Phytomedicine

The stem bark of *Croton gratissimus* has been extensively used in South African ethical communities to treat various ailments. It has been infused with milk to manage stomach and intestinal disorders in the Zulu community because of its purgative property [15, 18, 23]. Unspecified uterine disorder was treated by blowing the powdered bark into the womb [15]. Powdered bark was rubbed into chest incisions to treat pleurisy [15]. In Nigeria, stem bark infusions were used to treat malaria [15]. The stem bark irritant property has been used on the chest wall in any painful respiratory condition (intercostal muscular pain), indigestion, dropsy, and neuralgia in Zulu community [23]. The stem bark was also used to manage earache, bleeding gums, skin inflammation, abdominal disorders, and chest pains [19, 24]. A combination of stem bark and roots was applied into incisions to manage swellings [19, 24]. The Owambo's community in Namibia used inner stem bark to prepare ear drops to manage earache or difficulty in hearing [23]. The stem bark decoction was used to treat insomnia and restlessness [23].

The leaves in South African communities were boiled, and steam bath was used to treat sores associated with sexual transmitted diseases [24]. Influenza, colds, fevers, and rheumatism were managed using smoked dried leaves and leaves in steam bath [23, 25]. In Zimbabwe and Botswana, the Shona community used leaf decoction with tea or leave smokes as cough suppressant [25, 26]. The leaf decoctions in Benin have been used as antihypertensive and antimicrobial (managing urinary tracts infections) agents [27, 28]. In Nigeria, malaria, dysentery, diarrhoea, convulsions, and diabetics have been treated and managed using leaves decoction [29, 30]. Fumes from a paste from grounded leaves with other two *Croton* species and goat fat when heated were used to treat insomnia and restlessness [23]. Eye disorders were also treated with leave decoction [25]. In Luhya and Agikuyu communities in Kenya, herbalists and tradition medicine men have used leaf decoction to provide wound healing regimens. Damara community used dried, crushed leaves as perfume and some boiled to produce fragrant soap [23].

Root decoctions in Zimbabwe communities has been used to treat aphrodisiac and abdominal pain [28, 30]. Menstrual pain and constipation in some Sudan communities have been treated with root infusions [29]. Kavango community used root and leaf extracts to prepare nose droplets to relieve cold and consume some to treat coughs [23]. Roots were grated into food as a tonic, especially for girls at the onset of their menses [23]. Finally, the Maa herbalists have used root and stem barks on Maasai communities in Kenya to treat respiratory disorders, diarrhoea, dysentery, syphilis, gonorrhea, and urinary tract infections. The ability of this plant to treat respiratory disorders is also supported through the work done by van Vuuren et al. studies [24]. Damara community used grounded dried roots as perfume [23].

## 4. Phytochemistry

*Croton* diverse species are a rich reservoir of phytochemicals as shown in various studies. These secondary metabolites have given *Croton* its medicinal importance over a long period of time. These phytoconstituents in *Croton gratissimus* include terpenoids, alkaloids, flavonoids, saponins, cardenolides, tannins, essential oils, phenolics and polyphenolics, steroids, carotenoids, indoles, organosulphur, and glycosides.

### 4.1. Secondary Metabolites

#### 4.1.1. Terpenoids

Terpenoids are derived from a 5-carbon atom isoprene (C_5_) which forms isoprene polymer known as terpene. Additional oxygen groups added to terpene forms terpenoids, also referred to as isoprenoid. Terpenoids are the highly dominant phytoconstituents in this genus. Isoprene (C_5_) units determine the groups of the terpenoid. The group hemiterpenoid (C_5_) has one isoprene unit, monoterpenoid (C_10_) has two isoprene units, sesquiterpenoid (C_15_) has three isoprene units, diterpenoid (C_20_) has four isoprene units, sesterterpenoid (C_25_) with five isoprene units, triterpenoid (C_30_) possesses six isoprene units, tetraterpenoid (C_40_) has eight isoprene units, and polyterpenoid (>C_40_) has more than 8 isoprene units [31].

*(1) Monoterpenoids (C_10_)*. Essential oils from *Croton gratissimus* leaves, stem, and roots extracts in Eastern Pretoria (South Africa) had monoterpenoids such as *α*-phellandrene, *α*-pinene, and Z- and E-*β*-ocimene [32, 33]. Other monoterpenoids in this species are linalool in *Croton gratissimus* and limonene and *ρ*-cymene found in *C. antanosiensis* and *Croton stellulifer*, respectively [32].

*(2) Sesquiterpenoids (C_15_)*. *C. zambesicus* leaf extracts had sesquiterpenes volatile oil (*β*-caryophyllene, *α*-copaene, and caryophyllene oxide). The root and stem bark essential oils yielded spathulenol, an oxygen tricyclic sesquiterpenoid [34]. *γ*-Cadinene and *α*-cadinene have been reported in *Croton geayi* [32]. Patchoulane (*C. muscicarpa*), guaiane (*C. regelianus*), bis-nor-sesquiterpenoids (*C. pedicellatus*), crocrassins A, B and cracroson H (*C. crassifolius*), and 1,3,5-cadinatriene-(7R,10S)-diol (*C. dichogamus*) were also validated [35].

*(3) Diterpenoids (C_20_)*. Diterpenoids are the major terpenoids in this genus with over 339 compounds validated [35]. They comprise of cembranoid, labdane, clerodane, kaurane, halimane, isopimarane, neoclerodane, phorbol, secokaurane, and trachylobane compounds. 10 new cembranolides were obtained from hexane, methylene chloride, and ethyl acetate *C. gratissimus* leaf extracts [25] (Table 2).

The stem bark extracts yielded six compounds, as given in Table 3 [36].

Dichloromethane leave extracts yield 8 diterpenes as given in Table 4 [37].

The labdane, crotonadiol, was isolated from *C. zambesicus* stem bark [38]. Diterpenoids (crotozambefuran, crotocorylifuran, and clerodanes) were also yielded. It also gave trachylobanes (7*β*-acetoxytrachyloban-18-oic acid and trachyloban-7*β*,18-diol) [37]. *Croton zambesicus* ethanolic leaf extracts yielded diterpenoids (*ent*-18-hydroxyisopimara-7,15-diene-3*β*-ol and 7*β*-acetoxytrachyloban-18-oic acid) [39].

*(4) Sesterterpenoid (C_25_)*. The only validated sesterterpenoid is pseudopulchellol from *C. pseudopulchellus* [35].

*(5) Triterpenoids (C_30_)*. Triterpenoids have been reported in *Croton* species with steroidal and pentacyclic structures. The stem bark ethanolic extracts of *Croton gratissimus* elucidated lupeol [32], and the methanolic extract of the dried fruit in North Africa yielded betulinic acid and botulin [40]. Lupeol, betulinic acid, botulin, and lupenone were also isolated from the seed of *Croton zambesicus* [41].

*(6) Tetraterpenoids (C_40_) and Polyterpenoids (C_>40_)*. Literature has mentioned the possible existence of tetraterpenoids and polyterpenoids within the genus and the *Croton gratissimus* species, but no study has yet to validate.

#### 4.1.2. Alkaloids

Alkaloids have been widely used ethnomedicinally for ages. It has a signature ammonia group in its structure making it alkaline.

The most frequent *Croton* alkaloids are compounds similar to benzylisoquinolines. They include aporphine, morphinandienones, tetrahydroprotoberberines, and proaporphine alkaloids. Glutarimide, guaiane, harman, nicotine, tyramine, and anabasine alkaloids have been isolated and validated from *Croton* species [32]. Crotamide and crotsparsidine (*Croton sparsiflorus*), crotonamide A (C. *pullei*), crotonine (*C. tiglium*), and crotonamide (*C. alienus*) have been also validated as alkaloids [35]. Different classes of alkaloids have been reported in this species, contributing to the medicinal importance of this genus.

#### 4.1.3. Flavonoids

Flavanols and flavones are in plenty in vascular plants. A common presence of flavanols and flavones in *Croton* species occurs as free highly methoxylated aglycones. Phenyl propanoids have been validated in *Croton* genus [42]. *C. gratissimus* methanol leaf extracts resulted to isolating tiliroside (kaempferol-3-O-*β*-6″(p-coumaroyl) glucopyranoside), isovitexin (apigenin-6-C-glucoside), and kaempferol. *C. zambesicus* ethanolic leaf extract also yielded the following alkaloids: quercetin (quercetin-3-O-*β*-6″(p-coumaroyl) glucopyranoside-3′-methyl ether (helichrysoside—3′-methyl ether)) and the two flavonoids stated above, that is, tiliroside and isovitexin [14]. *C. zambesicus* extracts have also validated vitexin alkaloids. The chloroform extracts of *Croton gratissimus* fruits yielded the following flavonoids: ayanin, laudanine, quercetin-3,3′,4′-trimethylether, quercetin-3,4′-dimethylether, retusin, quercetin-3,7-dimethylether, naringenin, 3-methoxy-4-hydroxybenzoic acid, and laudanosine. These compounds have shown antiprotozoal activity [43]. Other flavonoids validated in other *Croton* species include ayanin (*C. schiedeanus*), rutin (*C. menthodorus*), taxmarixetin, and eriodictyol (*C. steenkampianus*) [32]. A flavone, crotoncaudatin, was isolated from *C. cauatus* [35]. More studies are needed to establish flavonoids in *Croton gratissimus.*

#### 4.1.4. Essential Oils

*Croton gratissimus* is a rich source of both fixed and volatile oils. These oils are majorly made up of diterpenoid, sesquiterpenoids, and triterpenoids. Some of them include *α*-cubebene, *δ*-cadinene, germacrene D, *α*-humulene, pentyl benzoate, borneol, terpinolene, copaene, *β*-elemene, *β*-eudesmol, and bicyclogermacrene among many [44]. *Croton zambesicus* roots, leaves, and stem barks have shown to be rich in essential oils constituted by monoterpenes and the root barks have sesquiterpenes [36]. Compounds such as shikimate derivatives, monoterpenoids, and sesquiterpenoids have been isolated and validated from volatile oils extracted from this genus [37].

The leaf extracts of *Croton zambesicus* contains essential oils such as linalool, *β*-caryophyllene, and p-cymene. The flowering top of this plant yielded pinene, linalool, carvone, limonene, thymol, cis-nerolidol, and *α*-humulene as essential oils [45].

#### 4.1.5. Glycosides

The leaves of *C. zambesicus* validated a flavone glycoside (helichrysoside-3′-methylether). Root ethanolic extract of *Croton zambesicus* have been reported to have reducing sugars and cardiac glycosides [46]. 13 megastigmane glycosides were validated with 7 from *C. cascarillodes* (crotonionosides A–G) and 6 from *C. oblongifolius* (oblongionosides A–F), diglyceride galactoside (sparsioside) from *C. sparsiorus*, and clerodane isocrotofolane glucosides from *C. limae* [35].

#### 4.1.6. Phenolic Compounds

These compounds have an aromatic ring attached to a hydroxyl group or more. They include coumarins, phenolic acids, lignans, isoflavonoids, flavonoids, phenolic polymers, and stilbenes. The leaves of *Croton gratissimus* elucidated phenolic compounds as shown in Lawal et al.' study [44].

*(1) Lignin*. A dihydrobenzofuran lignan 3′,4-*O*-dimethylcedrusin, a lignoid with a benzylisoquinolines structure, has been elucidated from *Croton* species. To confirm that *Croton* species, leaf and stem bark extracts have lignoid; these extracts were immersed in 10% phloroglucinol for 15 min, mounted in 25% hydrochloric acid, and analysed microscopically, lignin-stained pink/red [42]. *C. kongensis* twigs and leaves extracts had Nor-lignan (8S-(−)-8-(4-hydroxy-3-methoxybenzoyl)-dihydrofuran-8(8′*H*)-one) [35].

*(2) Carboxylated Polysaccharides, Free Phosphate Groups Macromolecules, Polyuronides, and Polyphenols*. *Croton gratissimus* leaves and stem bark extracts were evaluated for the above isolates by placing them in 0.05% toluidine blue for 1 min. They were rinsed with distilled water. Carboxylated polysaccharides, free phosphate groups macromolecules, and polyuronides stained purple and polyphenols (lignins) stained green/bright blue [42].

*(3) Proanthocyanidins*. Proanthocyanidins are polyphenols (tannins) rich in plants and have medicinal properties. Proanthocyanidins (tannins) have been validated in *Croton zambesicus* [42]. Tannins precipitate microbial proteins by complexing irreversibly with proline, making nutritional vital proteins unavailable for microbial growth. This causes microbial cell protein synthesis inhibition [47].

#### 4.1.7. Other Compounds

Root ethanolic extract of *Croton zambesicus* have been reported to have saponins and anthraquinones [46]. The ethanolic leave extract also reported the presence of saponins [48].

Coumarin, phenylbutanoids, polyprenols, amino acids, wax esters, furanoarabinogalactan, a polysaccharide, and cyclopeptides have been suggested to be in *Croton gratissimus* [35].

## 5. Pharmacological Activity

### 5.1. Antimicrobial Activity

#### 5.1.1. Antibacterial Activity

Essential oils from the aqueous leaf extracts of *Croton gratissimus* yielded major compounds such as sabinene, *α*-phellandrene, *β*-phellandrene, *α*-pinene, and germacrene D, which exhibited bacteriostatic and bactericidal activities. The activity was stronger against Gram-positive bacteria with *Staphylococcus aureus* and *Staphylococcus faecalis*. It also had a slightly stronger activity against *Escherichia coli*. *Proteus vulgaris* (CSIR), *Klebsiella pneumoniae,* and *Pseudomonas aeruginosa* had moderate activity. Both *Proteus vulgaris* (ATCC) and *Enterococcus cloacae* had the weakest activity. All these activities were compared with the corresponding MICs of chloramphenicol and gentamycin [44].

Ethanol leaf extracts of *Croton gratissimus* had sensitivity to Gram-positives *Bacillus subtilis and Staphylococcus aureus* and Gram-negative *Escherichia coli* and *Pseudomonas aeruginosa* [49].

Methanolic leaf extracts of *Croton gratissimus* were evaluated for antibacterial activity against multidrug resistant *Staphylococcus aureus* and *Staphylococcus epidermidis.* It inhibited *Staphylococcus aureus* (5%, 4%) and *Staphylococcus epidermidis* (17%, 33%) at MICs of 0.2 *μ*g/ml and 0.6 *μ*g/ml, respectively [18].

The stem bark ethanolic extracts of *Croton zambesicus* validated antibacterial activity against Gram-negative *Klebsiella pneumoniae, Escherichia coli, Proteus mirabilis*, and *Pseudomonas aeruginosa.* It also has activity against Gram-positive *Bacillus megaterium, Bacillus subtilis,* and *Staphylococcus aureus* pathogens, although weaker compared to ampicillin and gentamycin activities [49].

#### 5.1.2. Antifungal Activity

*Croton gratissimus* ethanolic roots and leaf extracts had activity on *Cryptococcus neoformans* and *Candida albicans* [24].

The *Croton zambesicus* stem bark ethanolic extracts exhibited antifungal activity against *Aspergillus niger, Microsporum species,* and *Penicillium species* compared to a tioconazole standard, although extracts' activity was weaker [50].

*Candida albicans* was sensitive to *C. hutchinsonianus* extracts. The root extracts of *C. bonplandianum* rich in ursane triterpenoids had activity against *Calletotricheme camellie, Fussarium equisitae, Curvularia eragrostidies, Alternaria alternata,* and *Colletorichum gloeosporiodes* fungi [35].

#### 5.1.3. Antiprotozoal Activity

*Croton gratissimus* fruits chloroform extracts had flavonoids: ayanin, naringenin, retusin, laudanine, laudanosine, quercetins (quercetin-3,3′,4′-trimethylether, quercetin-3,4′-dimethylether, and quercetin-3,7-dimethylether), and 3-methoxy-4-hydroxybenzoic acid. These compounds showed various antiprotozoal activities [51].

*(1) Antileishmanial Activity*. The flavonoids were tested for activity against axenic amastigote *Leishmania donovani* and intramacrophage amastigote *Leishmania donovani* with miltefosine drug as a positive control. Quercetin-3,7-dimethylether and ayanin evoked highest sensitivity against axenic amastigote *Leishmania donovani.* Quercetin-3,3′,4′-trimethylether and quercetin-3,4′-dimethylether had moderate leishmanial activity against axenic amastigote *Leishmania donovani*. Laudanine and laudanosine yielded marginal activity. Intramacrophage amastigote *Leishmania donovani* showed no activity when these compounds were tested [43]. This was probably due to the interference of cellular permeability by these compounds by binding to cytosolic proteins or they are metabolized in the host cell phagolysosomes [52, 53].

*Croton zambesicus* roots ethanolic extracts and its fractions (butanol, hexane, ethyl acetate, dichloromethane, and aqueous) from Urua Area, Akwa Ibom State, Nigeria, were evaluated for *antileishmanial* action using cultured *Leishmania major* (Desto) promastigotes. The standards were pentamidine and amphotericin B drugs. They had antileishmanial properties against *Leishmania major*. The ethanolic extract (ED_50_, 58.18 *μ*g/ml) and ethyl acetate fraction (ED_50_, 51.10 *μ*g/ml) exhibited more activity than the other fractions (ED_50_ > 100 *μ*g/ml), although not comparable to the standards pentamidine (ED_50_, 5.09 *μ*g/ml) and amphotericin B (ED_50_, 0.29 *μ*g/ml) [41]. The root extracts have terpenes, palmitic acid, hexadecenoic acid, hexadecenoic acid, linoleic acid, and ethyl ester that provide antileishmanial activity [30, 41].

*(2) Antitrypanosomial Activity*. These compounds elucidated in *Croton gratissimus* ethanolic leaf extracts were evaluated for in vitro trypanosomiasis activity against *Trypanosoma brucei rhodesiense* (STIB 900) blood stream against melarsoprol drug as the standard. Quercetin-3,3′,4′-trimethylether was active with high selectivity, quercetin-3,4′-dimethylether was potent with low selectivity, and ayanin was active but not selective. No activity was observed with retusin, naringenin, laudanine, and laudanosine [43].

*(3) Antiplasmodial/Antimalarial Activity*. This antiplasmodial activity of *Croton gratissimus* compounds was tested on *Plasmodium falciparum* with chloroquine as the positive control. Ayanin and quercetin-3,7-dimethylether exhibited activity with quercetin-3,7-dimethylether showing high selectivity than ayanin. Quercetin-3,4′-dimethylether showed activity, while 3-methoxy-4-hydroxybenzoic acid was with the least activity [43].

The ethanolic root extracts and fractions (chloroform, ethylacetate, hexane, and methanol) of *Croton zambesicus* also demonstrated significant antiplasmodial activity against *Plasmodium berghei berghei* which were comparable to the standards, chloroquine and pyrimethamine drugs. The ethanolic extracts had a dose-dependent chemosuppression effect on the parasite (at 54 mg, 85.8% and 81 mg, 86.18%) against chloroquine 5 mg/kg, 83.91% reducing parasitaemia. Ethanolic extracts' prophylactic effect (at 27 mg/kg, 69.24%; 54 mg/kg, 77.74%; and 81 mg/kg, 79.34%) is against the standard pyrimethamine 1.2 mg/kg, 78.96%. The median and highest extract doses reduced parasitaemia comparable to the standard pyrimethamine. The curative test also elucidated a dose-dependent parasitaemia reduction at ethanolic extract doses (27 mg/kg, 17.66%; 54 mg/kg, 13.50%; and 81 mg/kg, 9.66 parasitaemia reduction) against chloroquine 5 mg/kg, 8.33% parasitaemia reduction with the negative control (distilled water 0.2 ml/kg) yielding 95.8% parasitaemia increase. The antiplasmodial chemo suppression activity at a dose of 54 mg/kg for each fraction of chloroform (75.39%), ethyl acetate (76.89%), and methanol (77.27%) was easily comparable. N-hexane fraction had the least activity at 57.88% [46]. Almost similar trend of results was observed; antiplasmodial activity was evaluated on ethanolic leaf extracts of *Croton zambesicus* [27]. *Croton gratissimus* Burch leaves' dichloromethane and methanolic extracts had good antimalarial activity against *P. falciparum* D10 strain [54]. Ajebesin et al. reported water-boiled *Croton zambesicus* leaves decoctions were used to treat malaria [55].

#### 5.1.4. Antiviral Activity

MTT assay (3-(4,5-dimethylthiozole-2-yl)-2,5-diphenyltetrazolium bromide) and anti-HIV assay determined the anti-HIV activity of the methanolic leaves extracts of *Croton gratissimus*. The standard was berberine, a known toxic compound to viruses. It was noted that 50% cytotoxic concentration (CC_50_) of *Croton gratissimus* methanolic leaf extracts (100 *μ*g/ml) was more than that of berberine (27 *μ*g/ml). The 50% effective concentration (EC_50_, 9.6 *μ*g/ml) and selectivity index (S.I, 10.4) of *Croton gratissimus* extracts were higher than for berberine (positive control), which did not record any value. These values of the methanolic extracts indicate anti-HIV activity [18]. Polyphenols (flavonoids), which have been isolated from *Croton gratissimus,* have been shown to contribute to antiviral activity. Tannins chelate to different points on the protein surface. Proanthocyanidin SP-303 had antiviral activity against a number of viruses. This has been validated against influenza A and B viruses, parainfluenza type-1 and type-3, respiratory syncytial virus, genital and anogenital herpes virus, HIV, and hepatitis viruses [37].

### 5.2. Antiplatelet Aggregation, Hemolysis, and Anticoagulant Activities

#### 5.2.1. Hemolysis

*C. zambesicus* ethanolic leaf extract' hemolysis properties of hemoglobin concentration (Hb), red blood cell (RBC) count, white blood cell (WBC) count, and packed cell volume (PCV) were evaluated and found to be reducing the above components. This was explained by hemolysis or ethanolic extracts, suppression of erythropoiesis, and leukocytosis and WBC production in the bone marrow [56]. Clarity about hemolysis was further tested with aqueous and dichloromethane leaf extracts of *Croton zambesicus*, and their fractions were subjected for analysis. No significant activity was observed with any extracts and fractions at the concentration of 1 mg/ml against Triton X-100 1% (v/v), the positive control with 100% hemolysis and PBS, and the negative control with 0% hemolysis [28].

#### 5.2.2. Antiplatelet Aggregation Activity

*C. gratissimus* leaf essential oil exhibited platelet agglomeration inhibitory properties and was concentration-dependent. The highest concentration (10 mg·L^−1^) gave a platelet accumulation inhibitory activity for adenosine diphosphate (69.0%), collagen (69.4%), epinephrine (54.2%), and thrombin (78.6%) compared to aspirin percentages of platelet aggregation inhibitory activity for adenosine diphosphate (61.0%), collagen (69.1%), epinephrine (57.6%), and thrombin (53.3%). The platelet accumulation initiated by thrombin, collagen, and adenosine diphosphate was more potent than that of aspirin. The lethal concentration (LC_50_ < 1 mg·mL^−1^) indicated that *C. gratissimus* essential oil had superior inhibitory of collagen-initiated platelet aggregation than aspirin at LC_50_, 4.2 mg·mL^−1^. Although epinephrine had the lowest ability to induce platelet aggregation LC_50_, 3.65 mg·mL^−1^ against the oil, it showed better potency of antiplatelet agglomeration activity than aspirin at LC_50_, 8.18 mg·mL^−1^ [44].

To further prove this activity, a test was done with human blood rich platelet plasma. *Croton zambesicus* leaf aqueous and dichloromethane extracts and their fractions at 200 *μ*g/ml dosage each did not significantly hinder platelet accumulation inhibitory activity induced by the four platelet agonists, 4 *μ*M adenosine diphosphate, 4 *μ*g/ml collagen, 5 *μ*M PAR1-AP, or 1 *μ*M U46619 [28].

#### 5.2.3. Anticoagulant Activity

*Croton zambesicus* leaf aqueous and dichloromethane extracts and their fractions dose dependently reduced maximum concentration (*C*_max_) and endogenous thrombin potential (area under the curve) inhibiting plasma clotting after a coagulation cascade was triggered by tissue factors or contact pathways. Aqueous extracts and fraction were more potent regardless of the coagulation triggers. These extracts and fractions at 100 *μ*g/ml inhibited amide cleavage activity of thrombin (THR), factor Xa (FXa), and tissue factor/factor VIIa complex (TF/FVIIa) enhancing anticoagulation property [28].

### 5.3. Anticholinesterase Inhibitory Activity

*C. gratissimus* ethyl acetate and butanol fractions yielded helichrysoside-3′-methyl ether, isovitexin, and tiliroside. Anticholinesterase inhibitory property was observed in isovitexin which was stronger compared to helichrysoside-3′-methyl ether and tiliroside. The activity of isovitexin may be attributed to the sugar moiety position creating room for ketone and hydroxyl groups to take part of the reaction readily. The extract compound does not react freely with tiliroside and helichrysoside-3′-methyl ether ketones and hydroxyl groups due to steric hindrance of the sugar moiety [14].

### 5.4. Antioxidant Activity

The four compounds (tiliroside, isovitexin, helichrysoside-3′-methyl ether, and quercetins) isolated from *C. gratissimus* methanolic extracts showed antioxidative properties through the following assays [14].

#### 5.4.1. DPPH Radical Scavenging Assay

DPPH (2,2-diphenylpicryhydrazyl) is a deep violet coloured stable molecule in methanol with an absorbance maximum of 515 nm. Antioxidants or free radicals react with DPPH by producing an electron or hydrogen atom, reducing it to 2,2-diphenylpicryhydrazine characterized by colourless or pale yellow colour. These compounds reduced the initial concentration of DPPH by 50% by changing its DPPH colour from deep violet to pale yellow, hence antioxidant activity [14, 47]. For effective antioxidant activity, the compounds needed to have catechol, unsaturated 3-OH, and keto groups. Quercetin and rutin compounds from the leaves of *Croton gratissimus* completely bleached DPPH because of the free scavenging radicals catechol, 3-hydroxyl, and keto groups present [57].

#### 5.4.2. *β*-Carotene-Linoleic Acid Model System (CLAMS)

This complex contains *β*-carotene giving it orange colour and linoleic acid. The complex is heated at 50°C to convert linoleic acid to linoleate free radical by removing the hydrogen atom. The linoleate free radical attacks the orange *β*-carotene to gain the lost hydrogen atom. In the process, *β*-carotene loses its orange colour. Bleaching of *β*-carotene is accelerated in the absence of an antioxidant. Ethyl acetate and dichloromethane extracts of *Croton gratissimus* prevented the bleaching of *β*-carotene, hence antioxidant activity [14].

#### 5.4.3. Ferric Reducing Power Assay

Ferricyanide is a yellow complex which can be reduced by a strong antioxidant to a greenish blue ferrous form with an absorbance of 630 nm wavelength. *Croton gratissimus* reduced ferricyanide from yellow to green blue. This was probably achieved through reacting with free radicals (ferricyanide) by giving electrons to create stable compounds terminating the chain reactions due to absence of free radicals [14].

#### 5.4.4. Phospholipids Peroxidation Assay Coupled with the Thiobarbituric Acid-Malondialdehyde (TBA/MBA) Prototype System

Lipid peroxidation produces a number of degeneration products such as malondialdehyde (MDA) that causes cell membrane decimination and cell damage. MDA is a secondary metabolite of oxidative lipid degeneration, an indicator of oxidative stress and lipid peroxidation. Low MDA indicates a higher protective ability. The methanol, hexane, and dichloromethane extracts of *Croton gratissimus* exhibited this property by yielding low MDA after a ferrous complex was coinduced in rat brain homogenates at the end of the reaction [14].

### 5.5. Antiproliferative/Anticancer/Antitumoral Activity

Nitric oxide has been shown to produce peroxynitrite after reacting with free radicals (superoxides). Peroxynitrite causes irreversible cell membrane damage and cell inflammation. This phenomenon promotes tumor growth and proliferation. Inhibition of nitric oxide production promotes antitumor growth and anticancer. Natural lipoxygenase inhibitors also promote anticarcinogenicity and antitumor growth. Free radicals contribute to the pathogenesis of tumors and cancer. Antioxidant compounds contribute also to inhibition of tumor growth and anticancer.

Some secondary metabolites found in *Croton zambesicus* ethanolic leaf extract like palmitic acid (hexadecanoic acid ester and n-hexadecanoic acid), linolenic (docosatetraenoic acid and octadecatrienoic acid), and unsaturated fatty acid have been reported to have anticancer and antitumor activities [58]. Isolated flavonoids and terpenes from the seeds and leaf extracts of *Croton zambesicus* have shown to have strong antioxidant activities. It is believed that these compounds could be in the roots of this plant and therefore contribute to its anticancer properties by eradicating free radicals [41].

*C. zambesicus* dichloromethane leaf extracts were evaluated for cytotoxicity properties against human cervix carcinoma cells, and they positively confirmed this activity. Trachylobane (*ent*-trachyloban-3*β*-ol) from *C. zambesicus* leaf extracts initiated concentration-dependent apoptosis in human promyelocytic leukemia cells [59]. A 2,12-cyclocembranolide, (+)-[1R^*∗*^,2S^*∗*^,7S^*∗*^,8S^*∗*^,12R^*∗*^]-7,8-epoxy-2,12-cyclocembra-3E,10Z-dien-20,10-olide elucidated from the stem bark hexane and methylene extracts of C. gratissimus exhibited moderate action against PEO1 and PEO1TaxR 9 (paclitaxel resistant) ovarian cancer cell lines [15].

The water, acetone, and ethanolic *C. gratissimus* leaf extracts were also evaluated for antiproliferative properties. It was to be achieved by testing anti-inflammatory, antioxidant, and antiproliferative activities [16].

Antioxidant property was evaluated using 2,2-diphenyl-1-picrylhydrazyl (DPPH) and 2,2′-azino-bis (3-ethylbenzothiazoline-6-sulfonic acid) (ABTS) methods. Nitric oxide (NO) and soybean 15-lipoxygenase (15-LOX) enzyme inhibition assays would achieve anti-inflammatory activity, while antiproliferative activity would be achieved by cytotoxicity assays using 4 cancerous cell lines (human cervix adenocarcinoma cells (HeLa), human epithelial colorectal adenocarcinoma cells (CaCO-2), human breast adenocarcinoma cells (MCF-7, 2), and human lung adenocarcinoma cells (A549) and a noncancerous cell (African green monkey (Vero) kidney cells) [16].

From Table 5, the lower IC_50_ values of *Croton gratissimus* ethanolic leaf extracts in both DPPH and ABTS exhibited potent antioxidant activity compared to the standards. Acetone extracts had moderate activity. Ethanolic extracts exhibited *Croton gratissimus* could be used to prevent oxidative stress. They also had inhibitory activity against nitric oxide and soybean 15-lipoxygenase enzymes, hence anti-inflammatory activity against quercetin anti-inflammatory standard, hence preventing cancer and tumor growths [16].

The cytotoxic activity of leaf extracts of *Croton gratissimus* to the different cell lines was evaluated using their lethal doses (LC_50_), 50% inhibitory concentration (IC_50_), and the selectivity index against a standard drug, doxorubicin. The results given in Table 6 were obtained.

The water, ethanol, and acetone *C. gratissimus* extracts were slightly cytotoxic against noncancerous cell lines (Vero cells) with LC_50_ of between 533.33, 462.88, and 152.30 *μ*g/ml. Although these extracts were cytotoxic to cancerous cell lines, they did not meet the threshold of in vitro cytotoxicity properties, since IC_50_ was more than 30 *μ*g/mL. These extracts also had higher selectivity index (SI) values compared to the standard doxorubicin. This indicated that the active compound in these extracts interact with specific cancer-associated receptors or molecules. Binding of these compounds to these specific cancer receptors or molecules activates a mechanism that results in cancer cell apoptosis. Due to low cytotoxicity and high selectivity index, a concentration-dependent apoptosis effector caspase-3 and caspase-7 enzyme activation on the four cancerous cell lines was carried out. The extracts activated the caspase-3 and caspase-7 enzymes, leading to apoptosis of the cancerous cell lines (HeLa, MCF-7, A549, and CaCO-2) [16].

### 5.6. Anti-Inflammatory Property

To determine anti-inflammatory properties of this plant, its ethanolic root extracts of *C. zambesicus* were subjected to carrageenin, egg albumin, and xylene-induced oedema. These models would establish how and what causes the inflammation. Carrageenin inhibits histamines, serotonins, and kinnins in the early stages of inflammation but did not inhibit prostaglandins that mediate the later stages of carrageenin-induced inflammation. Xylene initiates phospholipase A2 enzymes to hydrolyse cell membrane phospholipids that release arachidonic, a substrate responsible for proinflammatory mediators' prostaglandins and leukotrienes. Egg albumin mediates inflammation by inhibiting histamine and serotonin release. The controls used were ASA, a cyclooxygenase inhibitor of prostaglandins, NSAIDS that have a paraamino group that have analgesic and antipyretic, but has weak to none anti-inflammatory activity, and dexamethasone which inhibits phospholipase A2 [60].

Carrageenin-induced oedema was weakly reduced by root extracts. This was attributed possibly to sesquiterpenes which cause peroxidations that destroy the cell membranes and the inability to inhibit prostaglandins. Egg albumin-induced oedema consequently was minimally reduced due to the inability to inhibit histamines and serotonins release, possibly inhibiting eicosanoids and kinnins. Highest dose of extract roots on xylene-induced oedema was significantly reduced. This indicated that the root extract inhibits phospholipase A2 enzymes [60].

Nitric oxide and soybean 15-lipoxygenase (15-LOX) enzyme inhibition assays affirmed anti-inflammatory action of *Croton gratissimus* leaf ethanolic extracts [16].

### 5.7. Analgesic Property

To evaluate the analgesic effects of the ethanolic root extracts of *C. zambesicus*, acetic acid caused writhing, formalin initiated paw licking, and hot plate thermally caused pain in mouse models were used. Acetic acid causes inflammation and pain by causing cell membrane capillary permeability, formalin sets in neurogenic and inflammatory pain, and hot plate-induced pain elaborates narcotic involvement [60].

The ethanolic root extract analgesic effect is compared to how paracetamol inhibits centrally prostaglandin synthesis in the brain by inhibiting COX-3 but does not inhibit prostaglandin peripheral biosynthesis, hence no peripheral anti-inflammatory effects but has analgesic and antipyretic effects. Ethanolic root extract acts centrally like paracetamol by prolonging the heat reaction time indicating the involvement of the narcotic or opioid receptors [60].

### 5.8. Antipyretic Activity

Ethanolic root extract of *C. zambesicus* inhibited pyrexia initiated by yeast, dinitrophenol, and amphetamine. The extracts act centrally reducing pyrexia possibly by diminishing brain prostaglandin E_2_ concentration, especially in the hypothalamus. This could be achieved by its action on COX-3 or enhancing the production of vasopressin and arginine, the body's own antipyretic substances [60].

### 5.9. Antidiabetic/Anticholesteremic/Antilipidemic/Antihypertriglyceridemic Activity

Diabetes mellitus is a chronic condition as a result of insulin insufficiency. Insulin is an anabolic hormone that regulates the metabolism of glucose-producing components (carbohydrates, fats, and proteins) and glucose absorption through glycogenesis, lipogenesis, and glucogenolysis. Its insufficiency results in the abnormal metabolism of glucose. This results in type 1 diabetes symptoms: hyperglycemia, glycosuria, polyuria, hyperlipidemia, muscle wasting, vision impairment, increased thirst, slow wound healing, and body weight loss among others. In type 2 diabetes, hypertriglyceridemia, hyperlipidemia, and hypercholesteremia are part of the complications experienced.

#### 5.9.1. Antidiabetic/Hypoglycemic Activity

It was noted that mice pretreated with ethanolic leaf extract of *Croton zambesicus* significantly reduced the blood glucose levels compared to the streptozotocin-induced diabetic mice at the tail end of the experimental period. Blood glucose levels increased in diabetic mice that ethanolic leaf extract of *Croton zambesicus* was withdrawn [61]. This was emphasized by the study done on alloxan-initiated diabetic rats where a decrease in blood sugar levels was dose dependent of the ethanolic leaf extracts of *Croton zambesicus* compared to the standard drug, chlorpropamide. This activity could be the effects of phytochemistry (saponins, terpenes, flavonoids, cardiac glycosides, polysaccharides, tannins, steroids, glycoproteins, polypeptides, and alkaloids) of the leave extracts which have been reported to contribute to this activity [45].

#### 5.9.2. Antihyperlipidemic/Anticholesterolemic/Antihypertriglyceridemic Activity

Insufficient insulin has led to accumulation of triglycerides in the liver after being converted from fatty acids. This results in hypertriglyceridemia. Insulin deficiency also decreased low-density lipoprotein receptors and increased high-density lipoprotein receptors. This results in increased low-density lipoprotein (bad) cholesterol and a decrease in high-density lipoprotein (good) cholesterol. Hypertriglyceridemia, an increase and decrease in bad and good cholesterol, respectively, leads to atherosclerotic cardiovascular diseases such as heart attack and stroke. As such, regulation of insulin and glucose goes a long way in preventing these complications. *Croton zambesicus* leaf extracts have shown to lower the low-density lipoproteins, very low-density lipoproteins, cholesterols, and triglycerides, but increase high-density lipoprotein cholesterol. This was comparable to the standard drug used, glimepiride [61]. *Croton zambesicus* leaves can be used to manage diabetes and hyperlipidemia for low-density lipoprotein cholesterol and hypolipidemia for high-density lipoprotein cholesterols [45].

### 5.10. Antiulcer Activity

Prostaglandin stimulates mucus secretion coating the gastric mucosa and bicarbonate secretion maintaining blood flow. Inhibiting prostaglandin synthesis through the cyclooxygenase pathway by indomethacin or histamine could cause gastroduodenal ulceration. Ethanolic root and leaf extracts of *Croton gratissimus* (syn. *C. zambesicus*) reduces ulceration in a dose-dependent way better than cimetidine [48, 62].

The gastric mucosa is known to be irritated by ethanol. Ethanol is toxic to the gastric mucosa by damaging it and changing its permeability and reducing gastric mucus. Free radicals (superoxide anions and hydroperoxyl) are normally released when ethanol is metabolized. These factor to gastric mucosa ulceration. Significantly, the ethanolic roots and leaf extracts of *Croton gratissimus* (syn. *C. zambesicus*) immensely reduced the ulcerations more than the standard propranolol, although in a dose-dependent manner [48, 62].

Reserpine-induced gastric ulceration has been attributed to overstimulation of smooth muscles through the vagus nerve (vagotonic hypermotility) and gastric mast cells degranulation resulting in an increase of gastric acid secretion. Reserpine mobilizes superoxide and hydroxyl radicals, inhibits mucus secretion, and stimulates *β*-adrenoceptors to breakdown gastric surface mucus. This creates an environment for gastric ulceration. *Croton zambesicus* ethanolic root extract reduced ulceration induction in a dose-dependent model which was lower compared to the standard drug cimetidine [62].

### 5.11. Anticonvulsant Activity

Anticonvulsant property of ethanolic root extract of *Croton zambesicus* was carried out on pentylenetetrazol (PTZ) and picrotoxin-induced convulsions. The standard drug used was phenytoin. Pentylenetetrazol (PTZ) is used as an anticonvulsant, but at higher doses, it causes seizures. Its anticonvulsant activity is achieved by inhibiting gamma aminobutyric acid (GABA, a neurotransmitter) activity on the GABA_A_ receptors. GABA is a major inhibitory neurotransmitter. PTZ by inhibiting neurotransmission activity of GABA will attenuate and enhance convulsion, hence epilepsy. Picrotoxin exerted its effect by blocking the GABA_A_ receptor-linked chloride ion channels. Activation of GABA receptors by GABA enhances chloride ion conductance into the brain cells, inhibiting repetitive action potential generation exerting antiepileptic activity. Phenytoin blocks sodium ions entering into the brain cells preventing repetitive action potential and epilepsy. Phenobarbitone and diazepam antagonize PTZ-inducing convulsions by enhancing GABA transmission. The ethanolic root extracts of *Croton zambesicus* have shown to highly delay the onset of clonic and tonic seizures induced by both PTZ and picrotoxin by enhancing GABA neurotransmission compared to phenytoin. Both the extracts and phenytoin does not completely prevent convulsions and mortality [62].

### 5.12. Insecticidal Activity

The essential oil of *Croton gratissimus* contains *α*- and *β*-phellandrene and germacrene D that have insecticidal activity. Germacrene D showed insecticidal activity against mosquitos, while *α*-phellandrene inhibited caterpillars from feeding [44].

### 5.13. Antihypertensive Activity

Potassium chloride and noradrenaline were used to evaluate the vasorelaxant properties of dichloromethane leaf extracts of *Croton zambesicus*. Potassium concentration in smooth muscle depolarizes the cell membranes. This leads to activating the L-type voltage worked calcium channels in the plasma membrane. Calcium channels open increasing the intracellular calcium resulting in smooth muscles contraction. Noradrenaline binds to G-protein-coupled *β*1-adrenoceptors in the plasma membrane depolarizing the cell membrane. This causes the calcium channels to open and increase intracellular calcium yielding smooth muscles contractions. Potassium and noradrenaline induced contractions increase cytosolic calcium. Verapamil (dihydropyridine derivatives), as the standard, inhibited voltage-operated contractions [63].

*Croton zambesicus* dichloromethane leaf extracts yielded *ent*-18-hydroxy-trachyloban-3-one and *ent*-trachyloban-14,15-dione compounds that were evaluated for vasorelaxant activity. These compounds significantly inhibited potassium-induced than noradrenaline-induced smooth muscles contraction in a dose-dependent manner [63].

*Croton zambesicus* dichloromethane leaf extracts yielded *ent*-18-hydroxy-trachyloban-3*β*-ol and *ent*-18-hydroxyisopimara-7,15-diene-3*β*-ol compounds that were subjected to vasorelaxant activity evaluation. Marrubenol (a diterpene) and verapamil were potent standards. The two compounds blocked KCl-induced contraction dose dependently. The mixture of these two compounds was three times potent than marrubenol and verapamil [64].

## 6. Toxicity

### 6.1. Antitumoral Growth/Carcinogenicity

Mutagenicity has been found to be a great tool in identifying anticancer agents. *Croton gratissimus* leaf extracts have shown this property against a number of cancerous (A549, CaCO-2, HeLa, and MCF-7) and noncancerous cell lines and African green monkey (Vero) kidney cells line [16]. Cytotoxicity has also been observed on ovarian cancerous cell line [15]. The extracts have also shown to suppress carcinogenicity and tumor cell growth [15, 16].

### 6.2. Hepatotoxicity

Hepatotoxicity risk evaluation was done on C3A and Vero cells applying various end point parameter analyses. Some of the markers such as mitochondrial membrane potential, mitochondrial mass, oxidative stress, lysosomal content, and lipid accumulation were used to assess the hepatotoxicity risk. *C*. *gratissimus* extract significantly diminished Vero and C3A cell density. It was also noted that a potent dose-dependent increase in lipid content and lysosomal compartment expansion identifies a potential risk, excess lipid within hepatocytes (steatosis) [65].

### 6.3. Genotoxicity

Genotoxicity risk was also examined with the help of micronucleus assay which showed a dose-dependent increase in micronuclei formation. Changes in nuclear morphology and cell ploidy further proved the associated genotoxicity risk and suggests the extract from *C*. *gratissimus* may function as an aneugen, affected cell division by interaction with the spindle apparatus and not directly by interacting with DNA [65].

### 6.4. Brine Shrimp Lethality Assay

A lethal concentration of 8.52 mg·mL^−1^ was obtained after a brine shrimp lethality assay was done. It clearly depicted that the essential oils of *C. gratissimus* are very toxic compared to the standard gallic acid with a lethal concentration (LC_50_) of 11.45 mg·mL^−1^ [44].

### 6.5. Acute Toxicity

The ethanolic leaf extracts of *Croton zambesicus* (100–200 mg/kg) produced physical toxicity signs ranging from reduced motor activity, writhing, reduced respiratory rate, and body and limb tone to death. The LD_50_ of the extract in mice was 1400 ± 148 mg/kg [45].

## 7. Conclusion

This literature review has shown that *Croton gratissimus* is of substantial ethnomedicinal importance having been used traditionally to manage various ailments such as cough, fever, pain, respiratory infections, abdominal disorders, and urinary disorders among others. The crude extracts of various plant parts have various pharmacological properties such as antimicrobial, anti-inflammatory, antipyretic, analgesic, antidiabetics, antiulcer, anticoagulant, antiproliferative, antioxidant, anticonvulsant, anticholinesterase inhibitory, and insecticidal properties. These pharmacological properties have been supported by the presence of vital phytochemicals such as terpenoids, phenolics, saponins, flavonoids, steroids, tannins, and alkaloids, although more studies need to be done for further elucidating their structures and identifying more phytochemicals. Despite the few toxicity studies done on this plant, more studies need to be done to validate the safety and efficacy of this plant, especially on its carcinogenicity and hepatotoxicity in humans.

## Figures and Tables

**Table 1:** Different names of *Croton gratissimus* [17, 22].

Community	Name
English	Lavender fever berry croton
Latin	*Croton gratissimus* Burch*Croton microbotryus* Pax*Croton antunesii* Pax*Croton welwitschianus* Müll. Arg*Croton zambesicus* Müll. Arg*Croton amabilis* Müll. Arg*Oxydectes amabilis* (Müll. Arg.) Kuntze*Oxydectes welwitschiana* (Müll. Arg.) Kuntze*Oxydectes zambesica* (Müll. Arg.) Kuntze
Shona	Gunukia, Mubangwa, Mufundemengwe
Afrikaans	Koorsbessie
Silozi	Mukena
Setswana	Mologa, Cassaca, Kanunkila
Damara	Gameb
Sudanese	Um-Geleigla
Gikuyu	Mukinduri
Swahili	Msuduzi
Luhya	Omuchindori/omusine
Kamba	Kithulu/muthulu
Samburu	Laeruguet or Lmarakweet
Maasai	Olokindigai or Enkitaru in Narok Ol-oiborbenek or Ol-ngergoit in Kajiado

**Table 2:** Diterpenoids (10 cembranolides) [25].

(−)-(1R^*∗*^,4R^*∗*^,10R^*∗*^)-4-Methoxycembra-2*E*,7*E*,11*Z*-trien-20,10-olide	(+)-(10R^*∗*^)-Cembra-1E,3E,7E,11Z,16-pentaen-20,10-olide
(+)-(1S^*∗*^,4R^*∗*^,8S^*∗*^,10R^*∗*^)-1,4,8-Trihydroxycembra-2E,6E,11Z-trien-20,10-olide	(+)-(5R^*∗*^,10R^*∗*^)-5-Methoxycembra-1E,3E,7E,11Z,15-pentaen-20,10-olide
(−)-(1S^*∗*^,4S^*∗*^,10R^*∗*^)-1,4-Dihydroxycembra-2E,7E,11Z-trien-20,10-olide	(−)-(1S^*∗*^,4R^*∗*^,10R^*∗*^)-1-Hydroxy-4-methoxycembra- 2E,7E,11Z-trien-20,10-olide
(−)-(1S^*∗*^,4S^*∗*^,7S^*∗*^,10R^*∗*^)-1,4,7-Trihydroxycembra-2E,8(19),11Z-trien-20,10-olide	(+)-(1S^*∗*^,4S^*∗*^,7R^*∗*^,10R^*∗*^)-1,4,7-Trihydroxycembra-2E,8(19),11Z-trien-20,10-olide
(−)-(1S^*∗*^,4S^*∗*^,10R^*∗*^)-1,4-Dihydroxycembra-2E,7E,11Z-trien-20,10-olide	(+)-(10R^*∗*^)-Cembra-1Z,3Z,7E,11Z,15-pentaen-20,10-olide

**Table 3:** Diterpenoids (6 cembranolides) [36].

(+)-(1R^*∗*^,10R^*∗*^)-Cembra-2E,4E,7E,11Z-tetraen-20,10-olide	
24-Ethylcholesta-4,22-dien-3-one	*α*-Glutinol
(+)-(1R^*∗*^,4S^*∗*^,10R^*∗*^)-4-Hydroxycembra-2E,7E,11Z-trien-20,10-olide	
4(15)-Eudesmene-1*β*,6*α*-diol	

**Table 4:** Diterpenoids [37].

*trans*-Phytol	*ent*-Trachyloban-3-one
*ent*-Trachyloban-3bol	*ent*-18-Hydroxy-trachyloban-3-one
*β*-Sitosterol	*α*-Amyrin
Isopimara-7,15-dien-3b-ol	Stigmasterol

**Table 5:** DPPH and ABTS assays show antioxidant activity and NO and 15-LOX show anti-inflammatory properties of *Croton gratissimus* leaf extracts against the standards' ascorbic acid, Trolox, and quercetin [16].

Plant	Extracts	50% inhibitory concentration IC_50_ in (*μ*g/mL)			
		DPPH	ABTS	NO	15-LOX
*Croton gratissimus*	Acetone	217.64	170.51	49.24	10.97
	Ethanol	32.18	34.95	51.93	2.58
	Water	>500	>500	88.90	>100

Positive control	Ascorbic acid	1.92	3.92	N/D	N/D
	Trolox	2.21	4.64	N/D	N/D
	Quercetin	N/D	N/D	5.82	24.60

**Table 6:** Activities of various leaf extracts of *Croton gratissimus* and standard (doxorubicin hydrochloride) on noncancerous and cancerous cell lines [16].

Extracts	LC_50_ (*μ*g/mL)	IC_50_ (*μ*g/mL) and selectivity index = LC_50_/IC_50_							
	Vero	A549	SI	CaCO-2	SI	HeLa	SI	MCF-7	SI
Acetone	462.88	97.46	4.75	74.05	6.25	78.21	5.91	83.74	5.52
Ethanol	152.30	79.60	1.91	48.46	3.14	73.78	2.06	39.75	3.83
Water	533.33	>200	<2.66	>200	<2.66	>200	<2.66	>200	<2.66
Doxorubicin	1.90	1.30	1.46	1.08	1.75	2.17	0.87	1.11	1.71

## Data Availability

The data used to support the findings of this study are included within the article.
